# Global Perspective on Kidney Biopsy and Glomerular Disease in North Macedonia

**DOI:** 10.34067/KID.0000001281

**Published:** 2026-06-09

**Authors:** Vlatko Karanfilovski, Nikola Gjorgjievski, Igor G. Nikolov, Pavlina Dzekova Vidimliski, Sabir Suleyman, Galina Severova, Lada Trajceska, Gjulshen Selim, Vesna Ristovska

**Affiliations:** Faculty of Medicine, University Hospital of Nephrology, Ss. Cyril and Methodius University in Skopje, Skopje, Republic of North Macedonia

**Keywords:** acute kidney failure, AKI, albuminuria, ANCA, CKD, chronic renal insufficiency, ESKD, glomerular diseases, GN, IgA nephropathy

## Overview of Kidney Disease and Nephrology Care in North Macedonia

North Macedonia is a Southeast European country with a population of approximately two million inhabitants and a surface area of about 25,700 km^2^.^1^ Glomerular diseases represent an important cause of ESKD in the country. Globally, the burden of CKD due to GN has markedly increased, with rises in incidence, prevalence, progression to kidney failure, and mortality, disproportionately affecting countries with lower sociodemographic status.^2^ According to the European Renal Association Registry, in 2023, glomerular diseases accounted for 13.1 incident cases per million population (pmp), representing 5.7% of patients initiating KRT, while the prevalence reached 134.7 pmp (13.8%) among patients receiving KRT.^3^ Nephrology care in North Macedonia is centered at the University Hospital of Nephrology in Skopje, founded in 1975 as the country's first and largest nephrology center. North Macedonia has approximately 20 nephrologists pmp, comparable with Western Europe (range, 9.47–55.75 pmp).^4^

## Kidney Biopsy Practice in North Macedonia

The first kidney biopsy in North Macedonia was performed in the 1970s at the University Hospital of Nephrology in Skopje, marking the beginning of histopathological diagnosis of glomerular diseases in the country.^5^ Today, the hospital remains the national center for kidney biopsies and management of glomerular diseases. Kidney biopsies are currently performed by four trained interventional nephrologists using an ultrasound-guided percutaneous technique. Rare, technically challenging cases requiring computed tomography guidance are performed by interventional radiologists. Studies from our center have shown that percutaneous kidney biopsy is a safe procedure, with complications observed in approximately 6% of 345 native kidney biopsies, while major complications occurred in 1.7% of cases (macroscopic hematuria requiring transfusion or intervention), and no biopsy-related mortality was reported.^6^ Over the past 10 years, only one biopsy-related nephrectomy has been documented at our center.

Immunofluorescence analysis of kidney biopsy specimens has been routinely performed at the University Hospital of Nephrology in Skopje for decades, enabling rapid preliminary diagnosis of glomerular diseases.^5^ The final diagnosis is established in Institute of Pathology in Skopje, where biopsy samples are evaluated using light and electron microscopy. Currently, only one experienced nephropathologist is available, with international collaboration used in challenging cases. In the last few years, kidney biopsies have also started to be performed at the General City Hospital 8th September in Skopje. According to data from the national kidney biopsy registry, a total of 89 kidney biopsies were recorded in 2025, of which 81 (91%) were performed at the University Hospital of Nephrology and eight (9%) at General City Hospital 8th September.

## Kidney Biopsy Activity and Spectrum of Glomerular Diseases in North Macedonia

Between 2016 and 2025, a total of 746 kidney biopsies were performed, including 710 native kidney biopsies (95.2%) and 36 biopsies in kidney transplant recipients (4.8%). The registry included all native and transplant kidney biopsies performed during the study period in patients aged 14 years and older from North Macedonia. Biopsies from foreign patients and children younger than 14 years, who are managed at pediatric centers, were excluded, while repeat biopsies were included. Kidney biopsy indications included nephrotic syndrome, proteinuria, impaired kidney function, and hematuria with or without proteinuria. Inadequate biopsies were defined as samples without kidney tissue or without glomeruli for histopathological evaluation. The presented cohort represents nearly all kidney biopsies performed in North Macedonia because the University Hospital of Nephrology and the General City Hospital 8th September are the only public hospitals where kidney biopsies are performed, while only a very small number are performed in private hospitals. Thirteen biopsies (1.7%) were inadequate. The number of biopsies varied across years, with the highest number in 2017 (*n*=121) and the lowest in 2020 (*n*=39), likely because of the coronavirus disease 2019 pandemic. All kidney biopsy specimens were analyzed by light microscopy. Immunofluorescence analysis was performed in 61.7% of biopsies. Available staining included IgA, IgG, IgM, C3, kappa, and lambda light chains. The lower proportion mainly reflected restricted use of immunofluorescence during the coronavirus disease 2019 pandemic, whereas outside this period, it was routinely performed in all native kidney biopsies and selectively in kidney transplant biopsies. Electron microscopy was available in 53.9% of cases during the last 5 years of the study period, increasing from 52.6% in 2021 to 60.8% in 2025, and was performed according to nephropathologist indication and local resource availability. The most common diagnosis was IgA nephropathy (12.9%), followed by membranous nephropathy (11.5%) and FSGS (10.3%; Table 1). The distribution of the most common glomerular diseases varied across the study period. IgA nephropathy remained the most frequent diagnosis overall, with a notable increase in 2025 (26.3%). Membranous nephropathy and FSGS were consistently observed throughout the study period without a clear temporal trend, while minimal change disease peaked in 2023 (16.7%) and crescentic GN was more common in 2021 (16.7%).

**Table 1:** Clinical and histopathological characteristics of kidney biopsies in North Macedonia (2016–2025)

Variable/Diagnosis	Value
**Overall cohort characteristics**	
Total kidney biopsies	746
Male sex	62%
Serum creatinine at biopsy (*µ*mol/L)	120 (81–196)
eGFR at biopsy (ml/min per 1.73 m^2^)	57 (33–95)
Proteinuria at biopsy (g/d)	2.1 (0.85–3.9)
**Most frequent biopsy-proven diagnoses, *n* (%)**	
IgA nephropathy	96 (12.9)
Membranous nephropathy	86 (11.5)
FSGS	77 (10.3)
Crescentic GN^a^	70 (9.4)
Minimal change disease	67 (9.0)

Clinical Characteristics by Diagnosis^b^					
Diagnosis	Age, Median (IQR)	Male, %	Creatinine (*µ*mol/L), Median (IQR)	eGFR (ml/min per 1.73 m^2^), Median (IQR)	Proteinuria (g/d), Median (IQR)
Membranous nephropathy	50.5 (37–68)	38.9	91 (64–139)	94 (37–102)	4.9 (3.0–7.3)
FSGS	49 (36–58)	20.7	114 (88–222)	54 (26–88)	2.6 (1.2–5.8)
Minimal change disease	37 (23–50)	34.5	86 (66–119)	98 (62–128)	3.0 (0.9–4.1)
IgA nephropathy	40 (31–53)	26.2	106 (80–150)	75 (44–100)	1.05 (0.38–1.83)
Crescentic GN^a^	64 (44–66.5)	25.0	358 (239–477)	17 (10–27)	1.35 (0.65–2.83)

IQR, interquartile range.

a Crescentic GN included both pauci-immune and immune complex–mediated forms.

b Among the remaining diagnoses, diabetic nephropathy and nephroarteriosclerosis (9.0%) were the most frequent, followed by mesangial GN without IgA deposits (7.9%), amyloidosis and fibrillary or immunotactoid glomerulopathy (6.9%), membranoproliferative GN (6.8%), lupus nephritis (5.5%), global glomerulosclerosis (3.4%), AKI (1.6%), and other diagnoses (5.7%).

Demographic and clinical characteristics according to the most frequent biopsy-proven diagnoses are presented in Table 1. Patients with minimal change disease were generally younger, whereas crescentic GN occurred in older individuals and was associated with the highest serum creatinine levels and lowest eGFR at the time of biopsy. Membranous nephropathy was characterized by the highest levels of proteinuria and nephrotic syndrome.

The distribution of biopsy-proven glomerular diseases in our cohort differs from earlier data from our center, where membranous nephropathy and mesangial proliferative GN were reported as the most frequent diagnoses, followed by IgA nephropathy and FSGS.^7^ In the present analysis, IgA nephropathy emerged as the most common diagnosis, likely reflecting improved diagnostic capabilities, broader availability of immunofluorescence and electron microscopy, evolving disease classification, and possible epidemiological changes over time. Similar patterns have been reported in large national registries. The Spanish Registry of GN, including more than 27,000 kidney biopsies, also identified IgA nephropathy as the most frequent primary glomerular disease, followed by membranous nephropathy and lupus nephritis.^8^ Comparable findings have been reported in biopsy series from Brazil, where IgA nephropathy, membranous nephropathy, and FSGS represent the leading diagnoses.^9^

## Conclusion and Future Perspectives

This overview provides contemporary insight into the epidemiology of biopsy-proven glomerular diseases in North Macedonia on the basis of data from the national kidney biopsy registry. Despite progress in nephrology care, several challenges remain. Expanding the number of centers performing kidney biopsies across the country could improve access to diagnostic procedures and allow earlier and more comprehensive evaluation of patients closer to their place of residence. Improving access to modern therapeutic options for glomerular diseases is another important priority, particularly with the emergence of new disease-modifying therapies that are not yet available in the country. Strengthening diagnostic capacity is also essential. Increasing the number of trained nephropathologists would improve the accuracy and timeliness of histopathological diagnosis. Systematic national recording of kidney biopsies and closer coordination between nephrology centers could support the development of a comprehensive national registry. Collaboration with larger international centers and participation in multicenter registries may further strengthen clinical research and improve the care of patients with glomerular diseases in North Macedonia.

## Supplementary Material
